# Primary care patients reporting concerns about their gambling frequently have other co-occurring lifestyle and mental health issues

**DOI:** 10.1186/1471-2296-7-25

**Published:** 2006-04-10

**Authors:** Felicity Goodyear-Smith, Bruce Arroll, Ngaire Kerse, Sean Sullivan, Nicole Coupe, Samson Tse, Robin Shepherd, Fiona Rossen, Lana Perese

**Affiliations:** 1 Department of General Practice and Primary Health Care, School of Population Health, The University of Auckland, Auckland, New Zealand; 2 Department of Social & Community Health, School of Population Health, The University of Auckland, Auckland, New Zealand; 3 Abacus Counselling & Training Services Ltd, Auckland, New Zealand

## Abstract

**Background:**

Problem gambling often goes undetected by family physicians but may be associated with stress-related medical problems as well as mental disorders and substance abuse. Family physicians are often first in line to identify these problems and to provide a proper referral. The aim of this study was to compare a group of primary care patients who identified concerns with their gambling behavior with the total population of screened patients in relation to co-morbidity of other lifestyle risk factors or mental health issues.

**Methods:**

This is a cross sectional study comparing patients identified as worrying about their gambling behavior with the total screened patient population for co morbidity. The setting was 51 urban and rural New Zealand practices. Participants were consecutive adult patients per practice (N = 2,536) who completed a brief multi-item tool screening primary care patients for lifestyle risk factors and mental health problems (smoking, alcohol and drug misuse, problem gambling, depression, anxiety, abuse, anger). Data analysis used descriptive statistics and non-parametric binomial tests with adjusting for clustering by practitioner using STATA survey analysis.

**Results:**

Approximately 3/100 (3%) answered yes to the gambling question. Those worried about gambling more likely to be male OR 1.85 (95% CI 1.1 to 3.1). Increasing age reduced likelihood of gambling concerns – logistic regression for complex survey data OR = 0.99 (CI 95% 0.97 to 0.99) *p *= 0.04 for each year older. Patients concerned about gambling were significantly more likely (all *p *< 0.0001) to have concerns about their smoking, use of recreational drugs, and alcohol. Similarly there were more likely to indicate problems with depression, anxiety and anger control. No significant relationship with gambling worries was found for abuse, physical inactivity or weight concerns. Patients expressing concerns about gambling were significantly more likely to want help with smoking, other drug use, depression and anxiety.

**Conclusion:**

Our questionnaire identifies patients who express a need for help with gambling and other lifestyle and mental health issues. Screening for gambling in primary care has the potential to identify individuals with multiple co-occurring disorders.

## Background

As available opportunities for gambling increase, it appears that problem gambling is increasing in prevalence [1]. Gambling disorders have been shown to have high comorbidity with the use of tobacco [1], problem drinking [2,3], other substance misuse [4], and mood disorder [5]. As well as impacting on an individual's health and well-being, problematic gambling may have serious harmful effects on the patient's family, financial security and career. Family physicians are often the first in the line to identify these problems and to provide a proper referral but problem gambling may go undetected during a standard consultation.

It is well known in the literature that comorbidity is linked with problem gambling and this link is bidirectional [6]. This connection between problem gambling and comorbidity has been widely supported worldwide mainly from treatment populations of problem gamblers, substance abusers, or psychiatric cohorts [7]. Within the general population, a link is reported between problem gambling and 'hazardous use of alcohol' as well as weaker associations between problem gambling and minor mental disorders and with substance abuse and psychiatric illness amongst young people [8]. Overall studies support the supposition that there is a link albeit a weaker one in the general population compared to treatment settings.

Comorbid conditions and problem gambling should not be viewed as discrete disorders, particularly when these individuals engage in treatment. Some problem gamblers will binge on alcohol if they do not have the resources to gamble [9]. Those with dual disorders may engage in other addictive behaviors such as alcohol or drug abuse when recovering from gambling, or relapse with gambling if they are also abusing substances [10].

Individuals with gambling and related comorbidity, tend to move in and out of these disorders. Many do not completely recover from these problem behaviors. For example, women casino employees were able to decrease the problem drinking symptoms over a three year time space frame, but they continued to gamble problematically [11]. Furthermore, many problem gamblers suffer from medical problems such as insomnia, irritable bowel syndrome, peptic ulcer, hypertension, migraines, and other stress-related problems which may be presented to the medical physicians rather than a gambling problem [12].

The aim of this study was to compare the group of New Zealand (NZ) screened primary health care patients who identified concerns with their gambling behavior with the total population of screened patients in relation to co-morbidity of other lifestyle risk factors and mental health issues.

## Methods

The assessment of the multi-item screening tool has been reported previously [13]. This is an instrument that contains screening questions for 10 potential issues: smoking, alcohol, substance abuse, gambling, depression, anxiety, stress, violence, eating disorders, physical activity. It also has the addition of a help question asking if the individual wants help no, yes, yes but not today. The gambling question *'Sometimes I've felt depressed or anxious after a session of gambling' *has been found to have a sensitivity of 0.857 and a specificity of 0.935 to a positive score in the EIGHT test for problematic gambling [14], which in turn has been validated against the South Oaks Gambling Screen (SOGS) [15]. Validity of the multi-item screening tool against a composite gold standard is currently underway.

The tool was assessed by 51 primary health care providers (family physicians or practice nurses) in one urban, one mixed urban and rural and one rural center in New Zealand. Practitioners were randomly selected using a computer-generated random number table. Multi-center ethical approval was obtained from the Auckland, Otago and Hawkes Bay ethics committees. Participant Information Sheets were provided both for practitioners and for patients, and written consent forms were signed from all participants.

Fifty consecutive adult patients were recruited per practitioner. All consecutive patients aged 16 years and over attending the practice (including those attending as caregiver of another patient) were invited to complete the lifestyle assessment screening tool and evaluation sheet. Exclusion criteria were patients who were unable to understand English or mental impairment that precluded meaningful participation. Demographic data included gender, age and ethnicity.

Data analysis, using descriptive statistics and non-parametric binomial (chi-squared tests and Fishers Exact 2-tailed) was conducted using SPSS-10.0 statistical package. Data included demographic information; positive responses to each screening question and number of patients requesting assistance from their doctor or nurse concerning risk factors.

The 79 who screened positive for concerns about gambling (answered yes to *'Do you sometimes feel unhappy or worried after a session of gambling?'*) were compared with the total patient population (2536) with respect to their responses to other screening factors. To examine the effects of age, gender, other behaviors on gambling status, a Pearson chi-squared statistic was corrected for the survey design using the second-order correction of Rao and Scott [16] and converted into an F-statistic. Adjusting for clustering by practitioner used STATA survey analysis, χ^2 ^and logistic regression (51 clusters). All analyses were done with the group of 79 as cases.

## Results

A total of 2,536 consecutive patients (1000 in Auckland; 1000 in Otago and 536 in Hawkes Bay), 20 urban doctors, 20 practice nurses and 11 rural doctors (51 practices) participated in the study. In Auckland, where patients were recruited by a research assistant, 23 patients actively declined to participate (97.75% response rate). In the other centers refusal rate was not formally recorded but research assistants said that it was less than 5%.

Forty-three of the 79 patients expressing concerns about gambling were female (54%), whereas two-thirds of total sample were female. Those worried about gambling more likely to be male with an odds ratio (OR) of 1.85 (95% CI 1.1–3.1).

The age of the 79 patients ranged from 18 to 89 years with a mean of 43 and SD of 16.3. When age was examined using logistic regression for complex survey data the OR = 0.99 (CI 95% 0.97–0.99) *p *= 0.035 for each year older – in other words, the older the patient, the less likely to identify as worried about gambling.

Maori (6%, 15/242) were significantly more likely than NZ European (1.55, 15/1002) to be worried about their gambling behavior (*p *= 0.0002) and were also more likely to want immediate help (*p *= 0.04) [17].

The group concerned about their gambling were also significantly more likely (all *p *< 0.0001) to have concerns about their smoking, use of recreational drugs, and alcohol (see Table 1). Similarly they were more likely to indicate a problem with depression, anxiety and anger control. They had no significant relationship for abuse, physical inactivity or weight concerns.

**Table 1:** Positive responses to screening questions (this is the odds of person being worried about smoking when also worried about gambling compared with the odds of all the group being worried about smoking)Total patients screened N = 2536 (from 51 practices); Patients worried about gambling n = 79 (3%)

	**Total N (%)**	**Worried about gambling n (%)**	*OR (CI 95%)	***p***
Do you ever feel the need to cut down on your smoking?*	406 (16)	30 (38)	3.9 (2.12 – 5.44)	<0.0001
Do you ever feel the need to cut down on your drinking?	258 (10)	18 (23)	2.74 (1.64 – 4.55)	<0.0001
Do you ever feel the need to cut down on your other drug use?	68 (3)	9 (11)	5.23 (2.51 – 10.9)	<0.0001
During the past month have you often been bothered by feeling down, depressed or hopeless?	1081 (43)	53 (67)	2.84 (1.7 – 4.75)	<0.0001
During the past month have you often been bothered by having little interest or pleasure in doing things?	805 (32)	42 (53)	2.5 (1.67 – 3.81)	<0.0001
Have you been worrying a lot about everyday problems?	997 (39)	46 (58)	2.21 (1.38 – 3.55)	<0.001
Is there anyone in your life whom you are afraid of, who hurts you in any way or prevents you doing what you want?	130 (5)	3 (4)	0.73 (0.24 – 2.24)	0.57
Is controlling your anger sometimes a problem for you?	387 (15)	24 (30)	2.52 (1.44 – 4.43)	<0.001
As a rule, do you do at least 30 minutes of moderate or vigorous exercise (such as walking or a sport) on 5 or more days of the week?	1379 (54)	47 (59)	1.24 (0.78 – 1.99)	0.36
Are you happy with your current weight?	1072 (42)	40 (51)	1.4 (0.88 – 2.25)	0.15

* Odds ratio for logistic regression taking into account clustering

The multivariable logistic regression with 'worry gambling' as the dependent variable is presented in Table 2. Because the responses to the two depression questions are highly correlated (0.47), only the first depression question was used in the model. The increased odds ratios for other factors for those concerned by their gambling show a risk picture of multiple and independent issues.

**Table 2:** Multivariable logistic regression with 'worry/gambling' as dependent variable

**'worry gambling'**	**OR**	**95%CI**
Cut smoking	2.86	1.83 – 4.47
Cut drugs	2.86	1.30 – 6.26
Depression (1st q)*	2.29	1.21 – 4.35
Male	1.85	1.11 – 3.07

*Answering 'yes' to 2 depression questions highly correlated (0.47)

Eleven out of the 79 (14%) who identified as having gambling concerns expressed a desire for help, five immediately and six at a later date. Those worried about their gambling were significantly more likely to want help with their smoking, other drug use, depression and anxiety (Table 3) but the small numbers means these results should be treated with caution.

**Table 3:** Patients wanting help with specific issue

	**Yes, today**		**Yes but not today**		**Yes, either today or later**			
	^a^All N (%)	^b^G n (%)	^a^All N (%)	^b^G n (%)	^a^All N (%)	^b^G n (%)	**^c^χ^2^**	***p***
Smoking	68 (3)	6 (8)	119 (5)	11 (14)	187 (7)	17 (22)	21.4	<0.001
Alcohol	10 (0.4)	1 (1)	19 (0.7)	0 (0)	29 (1)	1 (1)	Fishers exact	0.604
Other drugs	8 (0.3)	1 (1)	9 (0.4)	1 (1)	17 (0.7)	2 (4)	Fishers exact	0.110
Gambling	5 (0.2)	5 (6)	6 (0.2)	6 (8)	11 (0.7)	11 (14)	167.3	<0.001
Depression	144 (6)	7 (9)	146 (6)	9 (11)	270 (11)	16 (20)	7.3	0.007
Anxiety	149 (6)	10 (13)	139 (5)	9 (11)	288 (11)	19 (24)	11.9	<0.001
Abuse	23 (1)	1 (1)	22 (1)	2 (3)	46 (2)	3 (3)	Fishers exact	0.182
Anger	28 (1)	2 (2)	50 (2)	3 (4)	78 (3)	5 (6)	Fishers exact	0.103
Exercise	28 (1)	0 (0)	57 (2)	1 (1)	85 (3)	1 (1)	Fishers exact	0.259
Weight	73 (3)	6 (8)	105 (4)	9 (11)	178 (7)	15 (19)	16.1	<0.001

^a ^All = total number of screened patients, N = 2356

^b ^G = patients expressing concern about their gambling behaviour, n = 79

^c ^Significance of difference between all screened patients and those expressing concern about gambling wanting help either immediately or later.

Only a proportion of patients acknowledging a problem identified that they would like help with this, either immediately or at a later date. Of those identifying smoking, 44% wanted help, 16% immediately; for alcohol use, 13% with 4.6% immediately; other drug use 28% with 14% immediately, and for gambling, 16% identified that they would like help with this behavior, 6% immediately.

## Discussion

It is not surprising that co-occurring symptoms such as depression, anxiety, and substance use linked with worries about gambling. Data do suggest that problem gambling can be associated with non-gambling health problems [18,19]. Co-occurring conditions were frequently identified amongst a group of patients concerned with their gambling behavior, particularly young males [20]. It is estimated that youth and adult problem gamblers in community and clinical settings drink alcohol and consume other legal and illegal substances at several times the average population rates [21,22]. A United States national problem gambling survey found 10% of lifetime pathological gamblers alcohol-dependent compared to 1.1% of non-gamblers [23]. A significant number of patients concerned about their gambling were more likely to be apprehensive about their smoking, use of recreational drugs and alcohol. Problem gamblers' rates of smoking have been shown to increase when they gamble [24].

Co-occurring rates of pathological gambling and mental disorders have been examined. Pathological gamblers have been shown to be significantly more likely than non-gamblers to suffer from anxiety disorder [25], and phobias [26] In the present study, patients responding yes to the gambling screen commonly also responded positively to the questions about depression, anxiety and anger control. While the depression questions have already been validated [27], validation of those on anxiety and anger control is currently underway.

It has been reported that moderate to high percentages of adults seeking treatment for pathological gambling have comorbid alcohol and/or substance misuse disorders. [28-30]. In addition, elevated rates of problem and pathological gambling (usually 10% to 20%) are evident among adults seeking professional help for alcohol and other substance misuse/dependence disorders [29,31-33]. Patients in this study who expressed concerns about their gambling, were also significantly more likely to want help with their smoking, other drug use, depression and anxiety. It has been shown that addition of the help question increases the specificity of the two depression questions used in this study from 67% to 89% while maintaining a sensitivity of 96% [34].

Research suggests that due to issues such as shame and stigma, gamblers are most likely to first seek assistance for gambling-related problems from informal sources of help (their family and friends) and to develop a range of self-help strategies prior to seeking formal (professional) assistance [35]. It is possible that the distribution of when patients would like help with their gambling could be partially explained by the above preferences of help-seeking.

Major reasons suggested for not seeking treatment are the desire to handle the problem without help, negative attitude related to stigmatization of addiction problems and embarrassment and pride [36]. For services to be accessible, they must be sensitive to the target demographics. For example, despite inflated problem gambling rates, some ethnicities [35,37] and age groups (adolescents) [38], often do not access mainstream gambling help agencies.

It should be noted that other reasons for a low rate of desire for help with gambling might reflect not having a gambling problem, not identifying a gambling problem that is present, or having a past gambling problem that has been resolved. The latter is less likely however, given that the gambling question is framed in the present tense.

A strength of this study is that it is the first to report co-morbidity in both lifestyle behaviors and mental health issues in a general practice setting. A weakness of this study is that we cannot be specific about the response rates in some of the centers but believe it to be low and unlikely to over-estimate any morbidities. Each question is quite brief however we know from other work that asking for help for depression is associated with a positive predictive value of 48% for major depression [39]. A further limitation is that a single question was used to assess gambling behavior with no specific timeframe referenced. While individual brief questions may have been validated, the composite tool has not yet been fully validated against a complied gold standard, although this work in underway. Furthermore because the question asks about feeling worried or unhappy after gambling, the likelihood of co-occurrence with a generalized anxiety or depressive disorder is increased and a positive response to the question does not necessarily indicate a gambling disorder.

## Conclusion

While screening is recommended by some authorities for depression, alcohol problems and obesity, some thought needs to be give to considering screening for problem gambling in primary care [40]. There is a need for more research, particularly of a detailed nature with more structured assessments used in conjunction with screening items, to improve prevention and treatment strategies.

## Abbreviations

CI confidence interval

MIST multi-item screening tool

NZ New Zealand

OR odds ratio

## Competing interests statement

The authors declare that they have no competing interests. The complete independence of researchers from funders is declared.

## Authors' contributions

FG conceived of the study, participated in its design, co-ordination and analysis and drafted the manuscript.

BA participated in the design and analysis of the study and helped draft the manuscript.

NK participated in the design and analysis of the study and contributed to manuscript revision.

SS participated in the design of the gambling component of the study and contributed to manuscript revision.

NC participated in the design and analysis of the study and contributed to manuscript revision.

ST participated in analysis of the study and contributed to manuscript revision.

RS participated in analysis of the study and contributed to manuscript revision.

ST participated in analysis of the study and contributed to manuscript revision.

FR participated in analysis of the study and contributed to manuscript revision.

LP participated in analysis of the study and contributed to manuscript revision.

All authors read and approved the final manuscript.

## Pre-publication history

The pre-publication history for this paper can be accessed here:


